# Specification of excitatory neurons in the developing cerebral cortex: progenitor diversity and environmental influences

**DOI:** 10.3389/fncel.2014.00449

**Published:** 2015-01-12

**Authors:** Marcos R. Costa, Ulrich Müller

**Affiliations:** ^1^Brain Institute, Federal University of Rio Grande do NorteNatal, Brazil; ^2^Dorris Neuroscience Center and Department of Cell Biology, The Scripps Research InstituteLa Jolla, CA, USA

**Keywords:** cerebral cortex, excitatory neurons, progenitor diversity, neuronal specification, development

## Abstract

The mature cerebral cortex harbors a heterogeneous population of glutamatergic neurons, organized into a highly intricate histological architecture. Classically, this mixed population of neurons was thought to be generated sequentially from a seemingly homogenous group of progenitors under the influence of external cues. This view, however, has been challenged in the last decade by evidences pointing to the existence of fate-restricted neuronal progenitors in the developing neocortex. Here, we review classical studies using cell transplantation, retroviral labeling and cell culture, as well as new data from genetic fate-mapping analysis, to discuss the lineage relationships between neocortical progenitors and subclasses of excitatory neurons. We also propose a temporal model to conciliate the existence of fate-restricted progenitors alongside multipotent progenitors in the neocortex. Finally, we discuss evidences for a critical period of plasticity among post mitotic excitatory cortical neurons when environmental influences could change neuronal cell fate.

## Classes of excitatory neurons in the mammalian cerebral cortex

The mammalian cerebral cortex harbors a heterogeneous population of neurons, which has been classically divided into two major groups: spiny and smooth neurons (Shepherd, 2003). It is accepted that in the adult mammalian cerebral cortex spiny neurons are excitatory neurons, whereas smooth neurons are inhibitory neurons (Migliore and Shepherd, 2005; Petilla Interneuron Nomenclature et al., 2008). Spiny neurons are usually classified according to the lamina where their soma is located and by dendritic morphologies. The latter allows the identification of pyramidal neurons and spiny stellate neurons.

Pyramidal neurons can be distinguished by their long apical dendrite, are found in all cortical layers except layer I and represent the major output neurons of the neocortex. It is estimated that most projections from pyramidal neurons connect different cortical regions, whereas only 1 in 100 fibers would connect subcortical targets (Braitenberg and Schüz, 1991). Pyramidal neurons also participate in local circuitry, representing the major source of excitatory input to the area in which they are found. Based on the differences in connections, pyramidal neurons are further classified as projection neurons with long axons that connect different cortical regions or project to subcortical targets; and interneurons with short axons that most commonly project locally (Shepherd, 2003).

Spiny stellate neurons have several dendrites of similar lengths and are found exclusively in layer IV of the granular cortex, where they represent the major recipient of thalamic inputs. Different from pyramidal neurons, spiny stellate neurons project mostly locally to areas near their cell bodies, although some can occasionally project to more distant cortical areas (Shepherd, 2003).

Cortical projection neurons can be further classified by hodology in associative, commissural and corticofugal subtypes (Molyneaux et al., 2007). Associative projection neurons extend axons within a single hemisphere, whereas commissural projection neurons connect neurons in the two cortical hemispheres either through the corpus callosum or the anterior commissure. Cortifugal projection neurons send axons to target areas outside the cerebral cortex, such as the thalamus (corticothalamic neurons), pons (corticopontine neurons (CPN)), spinal cord (costicospinal neurons), superior colliculus (corticotectal neurons) and striatum (corticostriatal neurons).

Cortical neurons can also be classified according to their main sensory inputs, as for instance, visual neurons, olfactory neurons, auditory neurons, somatosensory neurons and gustatory neurons in primary sensory areas. In more complex sensory areas, neurons can be classified as bi-modal neurons (role in the processing of two different sensory modalities) and multi-modal neurons (role in the processing of many different sensory modalities). Physiological classes of cortical neurons can also be distinguished according to their electrical intrinsic properties (Connors and Gutnick, 1990). For instance, while regular-spiking pyramidal neurons are observed in cortical layers II to VI, intrinsically bursting neurons are restricted to layers IV and V (Connors and Gutnick, 1990). Another difference can be observed in interspike interval (ISI). Whereas layer IV neurons usually evoke a short burst of action potentials with ISI <40 ms, layer V neurons have a longer first ISI (De la Rossa et al., 2013).

Furthermore, there is correlation between the laminar position of cortical neurons and their connection patterns (Douglas and Martin, 2004). Commissural neurons, for example, are mostly found in layers II, III and V, whereas corticothalamic neurons tend to be located in layer VI and subcerebral neurons in layer V. Differences in the projection patterns of subtypes of cortical neurons have been ingeniously exploited by the Mackli’s laboratory to identify specific molecular features of neurons settled in different cortical layers of the mouse brain (Arlotta et al., 2005). Using microinjection of fluorescent microspheres into distinct axonal tracts, the authors retrogradely labeled three neuronal populations: corticospinal motoneurons (CSMN), callosal neurons (CN) and CPN. These cell populations were then isolated at four different developmental time points (E17, P3, P6 and P14) by fluorescent-activated cell sorting (FACS) and their gene expression profile was studied by gene-chip analysis. This approach led to the identification of genes involved in the specification, morphologic maturation and connectivity of layer V CSMN, such as Fezl (Fezf2) and Ctip2 (Arlotta et al., 2005; Chen et al., 2005, 2008; Molyneaux et al., 2005). Other studies have described genes involved in the specification of callosal projection neurons (Alcamo et al., 2008; Britanova et al., 2008) and corticothalamic neurons (McKenna et al., 2011).

Many other molecules are expressed in a layer-specific pattern in cortical neurons (Molyneaux et al., 2007) but their expression may not correlate with specific subtypes of excitatory neurons. Table 1 summarizes some of these neuronal molecules, which will be of interest for our following discussion. Importantly, however, expression of these molecules is frequently variable across different cortical areas, leading to three important caveats: (i) neurons from the same cortical layer not necessarily express the same molecular marker; (ii) lack of expression of a given layer-marker not necessarily means that neurons do not belong to that layer; and (iii) molecules expressed in a layer-specific manner into a particular cortical area can be expressed in a different fashion into another areas. These limitations are particularly important for the interpretation of experimental data, as we shall discuss in the next chapters.

**Table 1:** **Relative expression of transcription factors in neurons of different neocortical layers**.

	Layers II/III	Layer IV	Layer V	Layer VI	References
Cux1/2	+++	+++			Nieto et al. (2004), Zimmer et al. (2004)
Svet1	+++	+++			Tarabykin et al. (2001)
Satb2	+++	+	++	+	Alcamo et al. (2008), Britanova et al. (2008)
Lmo4	+++		+		Bulchand et al. (2003), Arlotta et al. (2005)
Brn2	+++		+		McEvilly et al. (2002), Sugitani et al. (2002)
RorB		+++			Schaeren-Wiemers et al. (1997)
Ctip2			+++	++	Leid et al. (2004), Arlotta et al. (2005), Chen et al. (2005)
Fezf2			++	+	Arlotta et al. (2005), Chen et al. (2005), Molyneaux et al. (2005)
Foxp2			+	+++	Ferland et al. (2003), Hisaoka et al. (2010)
Tle4			+	+++	Hack et al. (2007)
Er81			+++		Hevner et al. (2003), Yoneshima et al. (2006)

*+++ highly expressed; ++ expressed; + weakly expressed*.

## Generation of excitatory cortical neurons

Cortical neurons in the mammalian cerebral cortex are generated in a limited period of development, varying from days to months depending on the species. In humans, cortical neurogenesis starts at gestational week (GW) 5 and finishes around GW20 (Bystron et al., 2008). In rodents, neurogenic intervals are much shorter lasting from embryonic day (E) 13 to E21 in rats (Berry and Rogers, 1965; Bayer et al., 1991) and E11 to E19 in mice (Angevine and Sidman, 1961; Caviness, 1982; Takahashi et al., 1993). These periods were defined by the administration to pregnant females of molecules that are incorporated into DNA during the S-phase of the cell cycle, such as tritiated thymidine (TH^3^) or BrdU. Later, the neuronal fate and laminar position of cells labeled with such molecules was determined by auto-radiography (TH^3^) or immunohistochemistry (BrdU).

These population studies showed that neurons destined for different cortical layers are generated in a temporal sequence, such that deep layer neurons are generated before upper layer neurons. Although these experiments did not distinguish between excitatory and inhibitory neurons, it is widely accepted that excitatory cortical neurons of layers II to VI generally follow this inside-out pattern (Greig et al., 2013). However, it should be noted that this does not hold true when one analyzes the birth data of neurons with similar projection patterns. This is most obvious for corticocortical and callosal projection neurons, which share the expression of molecular markers and are located predominantly in layer II-III but are also found in substantial numbers in deep layers. These molecularly and functionally similar neurons are born over an extended time window ranging between ~E11.5 and E15.5 (Greig et al., 2013).

Two main progenitor populations in the dorsal telencephalon are responsible for the generation of cortical excitatory neurons: (i) ventricular zone (VZ) progenitors or radial glia cells (RGC); and (ii) subventricular zone (SVZ) or intermediate progenitors. VZ progenitors are the first cells in the developing telencephalon and generate SVZ progenitors and neurons (Takahashi et al., 1995; Malatesta et al., 2000, 2003; Miyata et al., 2001, 2004; Noctor et al., 2001, 2002, 2004). SVZ progenitors were first described as gliogenic (Takahashi et al., 1995), but later acknowledged as an important source of cortical neurons (Haubensak et al., 2004; Miyata et al., 2004; Noctor et al., 2004).

More recently, genetic fate-mapping experiments using the Cre/LoxP system have provided direct evidence for the generation of glutamatergic cortical neurons from a discrete population of progenitors located in the dorsal telencephalon (Gorski et al., 2002). By crossing Emx1-Cre transgenic mice to a Cre-reporter mouse, the authors could show that Emx1-expressing progenitors are confined to the dorsal telencephalon and contribute glutamatergic neurons to all cortical layers, but not GABAergic cortical neurons.

The contribution of subtypes of progenitors to subtypes of excitatory neurons in different cortical layers remained unknown until 10 years ago, when studies in mice suggested that SVZ progenitors could contribute preferentially to the generation of upper layer neurons (Tarabykin et al., 2001; Nieto et al., 2004; Zimmer et al., 2004; Sessa et al., 2008, 2010; Dominguez et al., 2013). However, SVZ progenitors are present in mice at early and late stages of cortical neurogenesis, and RGCs generate directly only 10% of all excitatory neurons in the cerebral cortex (Kowalczyk et al., 2009). Since IPCs are generated from RGCs (Haubensak et al., 2004; Miyata et al., 2004; Noctor et al., 2004), these two cell types likely represent different progenitor states along a developmental time line rather than separate fate-restricted lineages. Indeed, a recent fate-mapping study using a Tbr2-Cre mouse line show that Tbr2^+^ cells, i.e., SVZ progenitors, contribute neurons to all cortical layers (Vasistha et al., 2014). Thus, SVZ progenitors likely represent an intermediate stage between VZ progenitors and cortical glutamatergic neurons during all cortical development. As a consequence, expansion of the SVZ in primates could reflect a homogeneous amplification of cell numbers in the cerebral cortex, rather than a selective expansion of upper cortical layers during evolution (Smart et al., 2002; Martinez-Cerdeno et al., 2006; Hansen et al., 2010).

## Are progenitor cells specified to the generation of particular subtypes of excitatory cortical neurons?

Although our knowledge about the generation of cortical glutamatergic neurons from a population point of view dates from several decades, much debate still exists on the possible relations between individual progenitors or subpopulation of progenitors and the generation of specific subclasses of cortical glutamatergic neurons.

Pioneer experiments from the laboratory of Susan McConnell addressed the potential of cortical progenitors from different developmental stages by transplanting these cells iso- or heterochronically into the developing cerebral cortex (McConnell, 1988; McConnell and Kaznowski, 1991; Desai and McConnell, 2000). These experiments showed that when progenitors were isolated from animals at late stages of corticogenesis, during times when layers II and III are generated, and transplanted into the brain of animals of a similar age (isochronic transplantation), they generated neurons of layers II/III and astrocytes (McConnell, 1985, 1988), thus resembling the fate of endogenously generated neurons. Next, presumptive layer V/VI progenitors were transplanted into the brain of animals of later developmental age (heterochronic transplantation), when layer II/III neurons are generated (McConnell, 1988). Most transplanted cells (80%) failed to migrate out from the injection site and the remainder (20%) differentiated into neurons in layers V and VI (57%) and II/III (43%). Based on these findings, the author concluded that “at least a subpopulation of embryonically generated neurons appears to be committed to a deep layer fate prior to migration” (McConnell, 1988).

Next, similar heterochronic transplantation experiments of presumptive layer V/VI progenitors into brains of animals of later developmental age were done using cells isolated at different stages of the cell cycle (McConnell and Kaznowski, 1991). The authors showed that cells isolated in S-phase generated neurons for layers II/III, similar to host cells. In contrast, cells isolated at later stages of the cell cycle generated neurons for layers V and VI, thus resembling the behavior of progenitors at the time of isolation. Together, these experiments suggested that environmental cues are important to specify the laminar fate of cortical neurons, but progenitors have a time-window to respond to such cues.

Other experiments showed that the capacity of cortical progenitors to respond to external cues is reduced during development (Desai and McConnell, 2000). Progenitors isolated at the stage when layer IV neurons are generated and transplanted into animals of later stages adopt the same fate as neurons generated in the host brain from endogenous progenitors, i.e., layers II/III neurons. In contrast, when transplanted into animals of earlier stages when layer VI neurons are generated, cells migrated to layer IV, the position typical of their origin.

Collectively, these transplantation experiments suggest that (i) environmental cues are important to determine the laminar fate of glutamatergic neurons; (ii) specification occurs at the level of progenitors; (iii) early progenitors respond to late extrinsic signal, but not the contrary; and (iv) post mitotic neurons are specified according to the environment where they are generated and do not change layer identity when exposed to new extrinsic signals. More generally, these experiments are cited as evidence of restriction in the fate potential of progenitor cells over developmental age. However, the data are also consistent with the existence of multiple progenitors, but where early and late progenitors behave different in different environments such as the late environment is conductive for the survival and differentiation of late-stage progenitors, while only the early environment sustains early progenitors.

Cell culture systems were also employed to study the potential of cortical progenitors to generate neurons and macroglial cells (Costa et al., 2009). However, only one study has accessed the lineage-relations among neurons bearing molecular hallmarks of individual cortical layers (Shen et al., 2006). This work showed that isolated cortical progenitor maintain the timing for generation of neurons destined to different layers, i.e., deep layer neurons first and upper layer neurons later. However, the authors could not show that a single progenitor could generate clones with both deep and upper layer neurons. Instead, they show examples of clones containing only upper layer Cux2^+^ neurons alongside neurons that do not label for other markers used. Since the panel of layer-markers available at the time to label subtypes of neurons was very restricted (Foxp2, Tle4, Er81 and Cux1), data could be interpreted in at least two ways: (1) Progenitors for deep and upper layer neurons comprise two different populations; or (2) Single progenitors can generate deep and upper layer neurons, but the markers used could not reveal this phenomenon.

**Figure 1:**
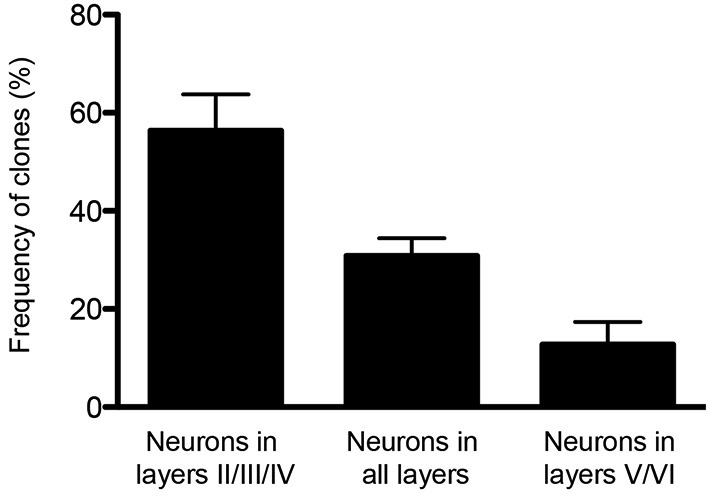
**Clones of spiny neurons, derived from neocortical progenitors labeled with retrovirus at E13, were classified according to the laminar position of clonally related neurons at P15**. The graphic shows the quantification of clones containing spiny neurons only in the upper cortical layers (II/III and IV), only lower cortical layers (V and VI) or both (*n* = 64 clones from 3 animals). Observe that 1/3 of clones contain neurons in both deep and upper layers.

Finally, the potential of single cortical progenitors to generate different types of cells was also assessed by retroviral-mediated fate mapping (reviewed by Costa et al., 2009). These studies rely on the infection of low number of progenitors and the identification of their progeny in the adult brain. Several technical issues complicate the interpretation of such studies, including silencing of retroviral vectors and labeling of GABAergic neurons that invade the cerebral cortex by tangential migratory routes (Costa et al., 2009). Nevertheless, fundamental insights into the behavior of progenitor cells and their contribution to the production of glutamatergic neurons of different cortical layers were derived from these studies (Luskin et al., 1993; Reid et al., 1995). In the first study, the authors described only one clone out of nine containing pyramidal (glutamatergic) neurons in both upper and deep layers (II and V) following retroviral injections at E15/16 rats. The other clones were restricted to layers II/III and IV (Luskin et al., 1993). Similarly, Reid et al. (1995) described clones containing pyramidal neurons either in deep layers (3 out of 15) or upper layers (12 out of 15) after retroviral injections of E15 rats. Using a combination of two retrovirus carrying plasmids encoding for green fluorescent protein (GFP) or red fluorescent protein (RFP) injected in E13 mice (Costa et al., 2009), we observed similar results, namely a bimodal distribution for clones of pyramidal neurons in upper or deep layers (Figure 1). Collectively, these experiments suggest that neurons destined to layers II-IV or V-VI tend to be generated from two different sets of progenitors.

## Lineage restricted progenitors for subtypes of neocortical projection neurons

Cre-Lox mediated genetic lineage tracing studies have recently provided new insights into the fate-potential of VZ progenitors. These lineage-tracing strategies depend on suitable genetic loci that allow for the expression of Cre in progenitor cells. Significantly, Cux2 is expressed in subsets of progenitor cells in the VZ (Franco et al., 2012) and SVZ (Nieto et al., 2004; Zimmer et al., 2004) already during earliest stages of neocortical development when layer VI and V neurons are born. At postnatal ages, Cux2 expression is most prominent in neurons within layers II-IV, but also found in subsets of neurons in deep layers (Nieto et al., 2004; Zimmer et al., 2004). The Cux2 expression pattern suggested a lineage relationship between Cux2^+^ progenitors and layer II-IV neurons. However, the co-existence of progenitors for late-born layer II-IV and early-born layer V-VI neurons early during cortical development is contradictory to a model of progressive restriction in the lineage potential of a common progenitor for all cortical projection neurons (Shen et al., 2006; Okano and Temple, 2009).

To carry out lineage-tracing studies, Cux2-Cre mice were generated by a knock-in strategy and crossed with several Cre-reporter mouse lines (Franco et al., 2012). The majority of labeled neurons (~75%) of the Cux2-Cre lineage were present in upper cortical cell layers of the mature cerebral cortex, but significant numbers (~25%) were also localized in deep layers. Consistent with the known expression pattern of Cux2 (Zimmer et al., 2004), many but not all of the neurons in deep layers were interneurons (Franco et al., 2012). Further analysis of the labeled projection neurons with molecular markers revealed that the majority expressed Satb2 (Franco et al., 2012), a marker for corticocortical projection neurons that are predominantly present in upper layers, but are also found in deep layers (Greig et al., 2013). Analysis of the expression of Ctip2, a marker for a subset of subcerebral projection neurons (Arlotta et al., 2005) revealed its expression in a small subset of neurons labeled by Cux2-Cre; some of them co-expressed Ctip2 and Satb2 (Franco et al., 2012). Thus, these data demonstrated that the majority of projection neurons in the Cux2-Cre lineage are Satb2^+^ cells.

Constitutively active Cre is an important read-out to reveal the full complement of cells expressing the gene under study at any time in a developing or mature tissue. Likewise, it allows identifying the cell types that do not express the gene under study. Thus, lineage tracing studies with Cux2-Cre revealed that a large fraction of Satb2^+^ cells but only very few Ctip2^+^ cells express Cux2 at any time during their developmental history. However, to define whether recombination occurred in progenitors, migrating cells and/or post mitotic neuron, temporal fate mapping studies are important. Therefore mice expressing tamoxifen-inducible CreERT2 from the Cux2 locus were generated, thereby conferring temporal control over Cre activity (Franco et al., 2012). The findings from these studies demonstrated that at E10.5 Cux2^+^ RGCs are specified to generate Satb2^+^ projection neurons in upper and deep layers of the neocortex. Importantly, further analysis demonstrated that Cux2^+^ progenitors are primarily proliferative during phases of lower layer neurogenesis and start to generate significant numbers of upper layer neurons only at later developmental time points. When the progenitors were forced to prematurely leave the cell cycle, they prematurely generated neurons expressing markers for upper layer neurons. Similarly, when progenitors were forced to differentiate *in vitro*, Cux2^+^ progenitors predominantly generated neurons expressing upper layer markers. Taken together, these findings suggest that a population of Cux2^+^ RGC cells is restricted in their fate potential to mostly generate Satb2^+^ projections neurons (Franco et al., 2012).

Recently, the model that Cux2^+^ progenitors are specified to generate Satb2^+^ projection neurons was challenged. Using Fezf2-CreERT2 mice, the authors proposed the existence of a multipotent progenitor for all neocortical projection neurons (Guo et al., 2013). Using the same tamoxifen inducible Cux2-CreERT2 mouse line used previously by Franco et al. (2012) the authors showed that neurons derived from the Cux2 lineage occupy at P0 both upper and deep neocortical cell layers (Guo et al., 2013). However, this result is expected since the formation of neocortical cell layers is not complete by P0. Many of the cells within the Cux2-lineage had at P0 the morphology of radially migrating neurons (Guo et al., 2013). In addition, Cux2-Cre traces not only Satb2^+^ cells in deep and upper cortical cell layers, but also a subset of interneurons especially in deep layers (Franco et al., 2012).

To further support their conclusion, the authors analyzed the phenotype of the neurons with molecular markers. These experiments were also carried out at P0 prior to the final maturation of cortical neurons. Many of the Cux2-CreERT2-derived projection neurons in deep layers express at P0 Ctip2, which is strongly expressed in layer V neurons that project to subcerebral targets (Arlotta et al., 2005). However, during early stages of differentiation, neurons frequently co-express genes that at later stages preferentially label subtypes of projection neurons with different layers position and projection patterns (Alcamo et al., 2008; Bedogni et al., 2010; Srinivasan et al., 2012; Deck et al., 2013). Furthermore, Ctip2 is also expressed at lower levels in other projection neuron subtypes (Arlotta et al., 2005), and the expression of other markers such as Satb2 was not evaluated. Thus the apparent discrepancy between the two studies is potentially explained by the fact that Guo et al. analyzed neuronal positioning and molecular phenotype during developmental time points with a limited set of markers, and markers such as Ctip2 where expression is not all-or-none but various in intensity between subtypes of neurons.

**Figure 2:**
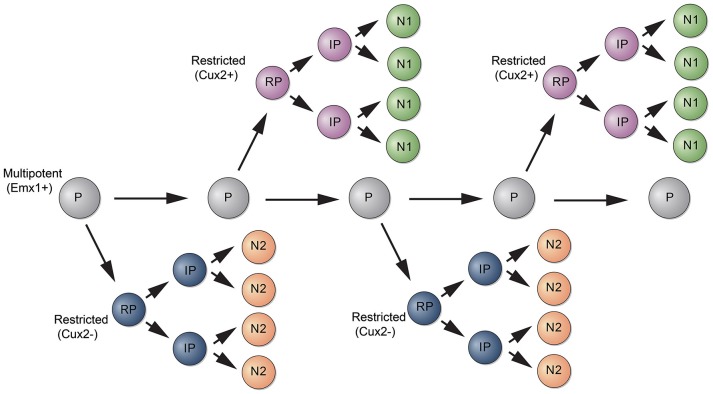
**Model for the generation of subtypes of projection neurons**. A multipotent progenitor (P, gray) persists in the cortical VZ and generates subtypes of restricted progenitors (RP, purple and blue). RPs generate intermediate progenitors (IPs) that differentiate into subtypes of neurons (N1, N2).

A recent study used a different strategy to analyze the potential of RGCs to generate neocortical projection neurons. The authors used Mosaic Analysis with Double Markers (MADM) to analyze the neuronal output from single RGCs (Gao et al., 2014). In MADM, Cre-recombinase induces interchromosomal recombination that reconstitutes fluorescent markers and allows tracing the progeny of progenitors where Cre was active. Using Emx1-CreERT2 mice and Nestin-CreERT2 mice, the authors induce recombination in progenitors between E10 and E13 and analyze their fate potential. Neurons in the Cre lineages occupied for the most part all neocortical cell layers. Studies with molecular markers suggested that most clones contain neurons with upper and deep layer identity. How can these data be reconciled with the findings from lineage studies using Cux2-Cre mice? Two points should be considered. First, the extent to which the MADM strategy is unbiased is unclear. Cre is active in a time window during mitosis and recombination thus likely depends on the length of the cell cycle of a particular progenitor and thus may not capture all progenitors. Perhaps more likely and interesting, Emx1-CreERT2 might label a multipotent progenitor that generates lineage restricted progenitors such as those labeled by Cux2-CreERT2 (Figure 2). This model is also consistent with retrovirus lineage tracing studies, which show that mulitpotent and restricted progenitors coexist within the cortical VZ (Figure 1).

Collectively, the new findings are consistent with a model where neuronal subtype specification occurs in part already at the level of progenitor cells. Perhaps, the cortical VZ is a mosaic of progenitors with different fate potentials, where a multipotent progenitor gives rise to lineage restricted progenitors (Figure 2). Of course, the cortex consists of many neuronal subtypes and there is heterogeneity even within each neocortical cell layer population (Britanova et al., 2008). Thus, further specification events are necessary to generate the immense diversity within neocortical projection neurons. Some of this diversity is established at the level of post mitotic neurons (Greig et al., 2013), suggesting that mechanisms acting both at the level of progenitors and post mitotic cells cooperate to generate subtypes of neocortical projection neurons.

## Plasticity of early-post mitotic neurons

Indeed, there are compelling evidences for an additional degree of plasticity at the level of post mitotic neocortical projection neurons. Both connectivity and electrical properties of neocortical neurons are affected by manipulations of sensory inputs after the neocortical neurogenic interval (Van der Loos and Woolsey, 1973; Sur et al., 1988), suggesting that the final fate of those neurons could be influenced by environmental cues acting at the level of post mitotic cells. Recently, Días-Alonso et al. have shown that conditional deletion of the cannabinoid receptor CB1 in post mitotic neurons decreases the numbers of Ctip2^+^ subcerebral projection neurons (Díaz-Alonso et al., 2012).

According to the notion that post mitotic neocortical projection neurons are plastic, two inspiring studies have shown that forced expression of a single transcription factor can shift fates of early post mitotic neurons (De la Rossa et al., 2013; Rouaux and Arlotta, 2013). Both groups have used genetic strategies to ectopically express Fezf2 in spiny neurons from layer IV (De la Rossa et al., 2013) and layers II/III (Rouaux and Arlotta, 2013), which in response acquired molecular identity, morphology, physiology and functional input-output connectivity of layer V projection neurons. Interestingly, the number of layer II/III neurons reprogrammed into layer V neurons by forced expression of Fezf2 is highest during the earliest stages of post mitotic differentiation (Rouaux and Arlotta, 2013). At later time-points, this plasticity decreases and is eventually abolished, suggesting the existence of a critical period of nuclear plasticity for post mitotic neurons. However, neuronal plasticity at late post mitotic stages can be partially rescued by combining Fezf2 expression with axonal sectioning (Rouaux and Arlotta, 2013). Together, these data indicate that cortical glutamatergic neurons retain some degree of plasticity, which is likely regulated by interplay between intrinsic transcriptional control and extrinsic network control (Russ and Kaltschmidt, 2014).

## Environmental signals controlling the generation of upper layer neurons

The temporal sequence for generation of neurons towards different cortical layers and/or lineages is also regulated by environmental cues. The neurotrophin Ntf3 acts as a feedback signal from post mitotic neurons to progenitors, promoting the generation of upper layer at the expense of deep layer neurons (Parthasarathy et al., 2014). Ntf3 gene is a target for Sip1, expressed at high levels in post mitotic neocortical neurons (Seuntjens et al., 2009). Conditional deletion of Sip1 in post mitotic neurons induces premature generation of upper layer neurons, also at the expense deep layer neurons (Seuntjens et al., 2009). However, down-regulation of Ntf3 produces an increase in layer VI neurons but does not rescue the Sip1 mutant phenotype, indicating that other signals are also involved in the control of cortical progenitor cell fate (Parthasarathy et al., 2014).

Genetic ablation of deep layer neurons also affects the fate of cortical progenitors, inducing *de novo* generation of deep layer neurons at the expense of upper layer neurons (Toma et al., 2014). However, it is not clear whether this effect is due to the lack of feedback signals from post mitotic neurons to progenitor cells, which would then resume generation of deep layer neurons, or by a direct fate conversion of post mitotic neurons. Future studies are needed to address the possibility of environmental signals contributing to the specification of neocortical projection neurons directly through regulation of transcriptional networks in both progenitors and post mitotic neurons.

## Conclusions

Generation of the large variety of neocortical spiny neurons starts at the level of progenitors in the neocortical VZ with the generation of at least two major classes of progenitors identified by expression or absence of Cux2. These progenitors are likely derived from a multipotent progenitor population and environmental cues may contribute for the establishment and balance of these populations.

Specification programs are inherited by post mitotic neurons and contribute to the laminar organization of the neocortex. The role of environment in the specification of neocortical spiny neurons at a post mitotic level requires more investigation, but is a potential mechanism to further refine the neocortical cytoarchitecture.

## Conflict of interest statement

The authors declare that the research was conducted in the absence of any commercial or financial relationships that could be construed as a potential conflict of interest.
